# Sociodemographic Predictors of SARS-CoV-2 Infection in Obstetric Patients, Georgia, USA

**DOI:** 10.3201/eid2611.203091

**Published:** 2020-11

**Authors:** Naima T. Joseph, Kaitlyn K. Stanhope, Martina L. Badell, John P. Horton, Sheree L. Boulet, Denise J. Jamieson

**Affiliations:** Emory University School of Medicine, Atlanta, Georgia, USA

**Keywords:** COVID-19, SARS-CoV-2, severe acute respiratory syndrome coronavirus 2, viruses, respiratory infections, zoonoses, coronavirus disease, Atlanta, Georgia, United States, infection, healthcare disparity, health status disparity, obstetrics

## Abstract

We conducted a cohort study to determine sociodemographic risk factors for severe acute respiratory syndrome coronavirus 2 infection among obstetric patients in 2 urban hospitals in Atlanta, Georgia, USA. Prevalence of infection was highest among women who were Hispanic, were uninsured, or lived in high-density neighborhoods.

Data from New York, New York, USA, have highlighted the disproportionate burden of coronavirus disease (COVID-19) in minority and low socioeconomic status communities in the United States (*1*), yet data are lacking from southeastern states, which are home to a large share of the nation’s racial and ethnic minority populations (*2**,**3*). The information is imperative in states like Georgia, which has average of 3,000–4,000 new cases daily and an urgent need for improved public health mitigation and containment strategies (*4*). The objective of this study was to determine associated sociodemographic and neighborhood risk factors for infection in an obstetric cohort undergoing testing for severe acute respiratory syndrome coronavirus 2 (SARS-CoV-2) in 2 urban hospitals in Atlanta, Georgia. Emory University School of Medicine Institutional Review Board and Grady Memorial Hospital Research Oversight Committee approved the study.

Universal testing for SARS-CoV-2 was initiated on all parturients admitted to labor and delivery at Grady Memorial Hospital, a safety-net hospital in Atlanta, starting on April 20, 2020, and at Emory University Hospital Midtown, an academic hospital in Atlanta, starting on April 24. Both hospitals serve a diverse, predominantly minority population; the safety-net hospital captures a larger proportion of uninsured and Medicaid patients (*5*). The safety-net hospital performs »2,500 and the academic hospital »5,000 deliveries annually. We abstracted test results, age, race, and ZIP code from electronic medical records and validated the data for all deliveries at both hospitals through July 29, 2020. Patient residential ZIP code was linked with data from the US Census Bureau to estimate median household income, proportion of households below federal poverty limit, use of public transit to travel to work, average household size, proportion of crowded households (>1 person per room), population density per square mile, neighborhood deprivation index, and proportion minority (nonwhite) residents (*2*). Data on neighborhood characteristics were derived from American Community Survey 5-year estimates for 2014–2018. Characteristics were calculated at the census tract level and linked to patient resident address using a validated ZIP code to tract crosswalk (*6*). To calculate the neighborhood deprivation index, we conducted principal components analysis using data for all census tracts in Georgia on 8 sociodemographic indicators, framed around domains of income, education, employment, and housing quality, producing a summary score ranging from −1.85 to 3.87, centered at 0; higher values indicated higher deprivation or more disadvantage.

We examined distributions of sociodemographic (age, race/ethnicity, and insurance status) and neighborhood characteristics by delivery hospital; we then combined data from both institutions to calculate prevalence rates and 95% CIs. For prevalence calculations, we dichotomized median income, proportion nonwhite residents, proportion below federal poverty limit, use of public transit, household crowding, neighborhood density, and neighborhood deprivation index by <50th percentile and >50th percentile. We performed data analysis using R (https://www.r-project.org); p values <0.05 were considered statistically significant.

We captured data on 1,882 women after delivery who had available test results, out of a total of 2,196 deliveries (Table; Appendix Figure). Overall SARS-COV-2 infection rate was 4.1% (77/1,882); prevalence was higher at the safety net hospital (9.4%, 58/616) than at the academic hospital (1.5%, 19/1,266). The study population was predominantly non-Hispanic Black (81.1%, 1,526/1,882) and publicly insured (43.9%, 826/1,882).

**Table:** Sociodemographic and neighborhood characteristics associated with increased prevalence of severe acute respiratory syndrome coronavirus 2 infection in laboring women, Georgia, USA*

Demographics	No. patients, N = 1,882	Total tested positive	Prevalence, % (95% CI)†	p value
Patient				
Age, y				0.34
<20	108	7	6.5 (1.8–11.12)	
20–35	1,393	57	4.1 (3.1–5.1)	
>35	381	13	3.4 (1.6–5.2)	
Race/ethnicity				<0.001
Non-Hispanic Black	1,353	52	3.8 (2.8–4.9)	
Non-Hispanic White	226	0	0	
Hispanic	139	22	15.8 (9.8–21.9)	
Asian/other	164	3	1.8 (−0.2 to 3.9)	
Insurance type				<0.001
Commercial	698	11	1.6 (0.7–2.5)	
Medicaid/Medicare	1,015	49	4.8 (3.5–6.1)	
Uninsured	169	17	10.1 (5.5–14.6)	
Neighborhood†				
Median household income				0.06
Lower average income	934	47	5.0 (3.6–6.4)	
Higher average income	939	30	3.2 (2.1–4.3)	
Below federal poverty limit				0.20
Lower poverty	924	32	3.5 (2.3–4.6)	
Higher poverty	949	45	4.7 (3.4–6.1)	
Use of public transit				0.25
Less transit use	950	34	3.6 (2.4–4.8)	
More transit use	923	43	4.7 (3.3–6.0)	
Proportion non-White				0.73
Lower percent minority	944	37	3.9 (2.7–5.2)	
Greater percent minority	929	40	4.3 (3.0–5.6)	
Household crowding				0.02
Small household size	944	49	5.2 (3.8–6.6)	
Large household size	929	28	3.0 (1.9–4.1)	
Neighborhood density				0.03
Less dense	911	28	3.1 (2.0–4.2)	
More dense	958	49	5.1 (3.7–6.5)	
Neighborhood deprivation index				0.64
Less deprived	935	36	3.9 (2.6–5.1)	
More deprived	938	41	4.4 (3.1–5.7)	

*Lower/less/small categories indicate <50th percentile; higher/more/large categories indicate >50th percentile. IQR, interquartile range; NDI, neighborhood deprivation index; Pr., proportion. †Prevalence is for entire cohort. Nine women provided out-of-state zip codes and were excluded from the neighborhood analysis; all 9 tested negative.

The prevalence of SARS-CoV-2 was highest among women who were Hispanic (15.8%, 95% CI 9.8–21.9; p<0.001) or uninsured (10.1%, 95% CI 5.5–14.6; p<0.001). Prevalence was also higher for women living in census tracts with smaller average household size (5.2%, 95% CI 3.8–6.6. vs. 3.0%, 95% CI 1.9–4.1; p = 0.02), increased neighborhood density (5.1%, 95% CI 3.7–6.5, vs. 3.1%, 95% CI 2.0–4.2; p = 0.03). We observed non–statistically significant increases in prevalence in census tracts with lower average household income, increased proportion of households below the federal poverty limit, and more neighborhood deprivation.

This study leveraged a universal SARS-COV-2 testing program in an obstetric cohort to determine sociodemographic and neighborhood risk factors for infection. Infection was significantly associated with Hispanic ethnicity, uninsured status, high neighborhood density. The counterintuitive findings for household size may be due to chance or the narrow range of average household size across included neighborhoods (1.5–3.8 persons/household) (*1*). 

This study has several limitations, including retrospective data collection and limited generalizability. However, its strengths include reporting of rates during a period of universal testing in an area outside of New York, and the use of census data to approximate neighborhood factors associated with increased risk for infection.

These data suggest that concentrated socioeconomic and neighborhood disadvantage, such as race/ethnicity, uninsured status, and neighborhood density, account for some of the disparate burden of COVID-19 illness in pregnant patients, a unique population at increased risk for perinatal illness (*7*). Research that considers specific features of neighborhood is needed to inform community-targeted mitigation strategies if viral containment is to be achieved.

Appendix Additional information about sociodemographic predictors of SARS-CoV-2 infection in obstetric patients, Georgia, United States.
