# Supplementary material for: Sociodemographic Predictors of SARS-CoV-2 Infection in Obstetric Patients, Georgia, USA
Source: Emerg Infect Dis. 2020 Nov;26(11):2786–8. doi: 10.3201/eid2611.203091 (PMC7588535; doi:10.3201/eid2611.203091)
Supplement: Appendix — Additional information about sociodemographic predictors of SARS-CoV-2 infection in obstetric patients, Georgia, United States. [file 20-3091-Techapp-s1.pdf]

Article DOI: https://doi.org/10.3201/eid2611.203091

Sociodemographic Predictors of SARS-CoV-2 Infection in Obstetric Patients, Georgia, USA

Appendix

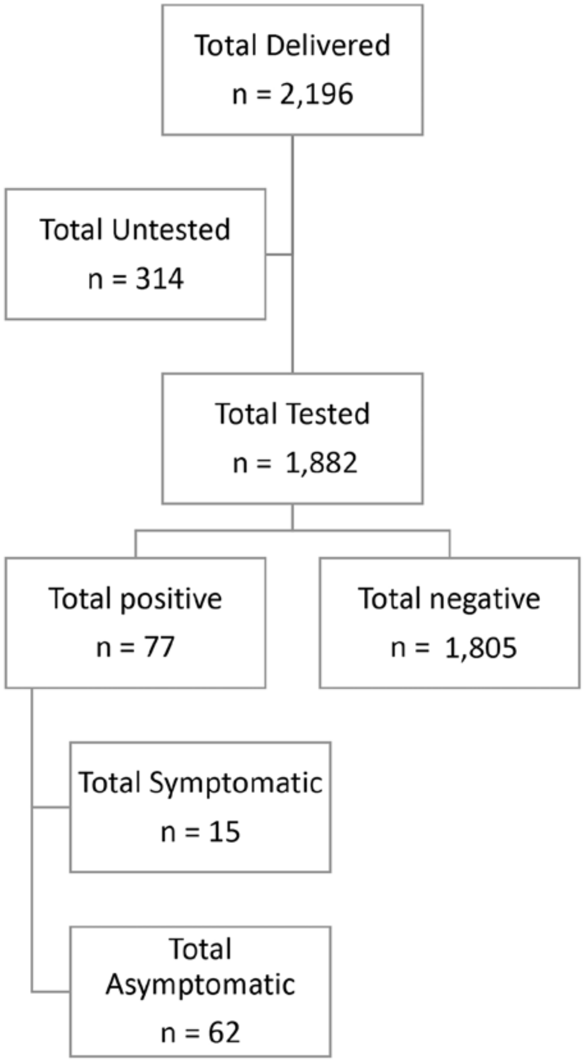

Appendix Figure. Flow diagram of laboring women in study of severe acute respiratory syndrome coronavirus 2 infection, Atlanta, Georgia, USA.
